# An Automated Prognostic Model for Pancreatic Ductal Adenocarcinoma

**DOI:** 10.3390/genes14091742

**Published:** 2023-08-31

**Authors:** Ioannis Vezakis, Antonios Vezakis, Sofia Gourtsoyianni, Vassilis Koutoulidis, Andreas A. Polydorou, George K. Matsopoulos, Dimitrios D. Koutsouris

**Affiliations:** 1 Biomedical Engineering Laboratory, School of Electrical & Computer Engineering, National Technical University of Athens, 9 Iroon Polytechniou St., 15780 Athens, Greece; ivezakis@biomed.ntua.gr (I.V.); dkoutsou@biomed.ntua.gr (D.D.K.); 2 2nd Department of Surgery, Aretaieion Hospital, School of Medicine, National and Kapodistrian University of Athens, 76 Vas. Sophias Ave., 11528 Athens, Greece; avezakis@med.uoa.gr (A.V.); apolyd@med.uoa.gr (A.A.P.); 3 1st Department of Radiology, Aretaieion Hospital, School of Medicine, National and Kapodistrian University of Athens, 76 Vas. Sophias Ave., 11528 Athens, Greece; sgty76@gmail.com (S.G.); vkoutoulidis@med.uoa.gr (V.K.)

**Keywords:** pancreatic ductal adenocarcinoma, pancreatic cancer, prognostication, survival, predictive modeling, radiomics, machine learning, deep learning

## Abstract

Pancreatic ductal adenocarcinoma (PDAC) constitutes a leading cause of cancer-related mortality despite advances in detection and treatment methods. While computed tomography (CT) serves as the current gold standard for initial evaluation of PDAC, its prognostic value remains limited, as it relies on diagnostic stage parameters encompassing tumor size, lymph node involvement, and metastasis. Radiomics have recently shown promise in predicting postoperative survival of PDAC patients; however, they rely on manual pancreas and tumor delineation by clinicians. In this study, we collected a dataset of pre-operative CT scans from a cohort of 40 PDAC patients to evaluate a fully automated pipeline for survival prediction. Employing nnU-Net trained on an external dataset, we generated automated pancreas and tumor segmentations. Subsequently, we extracted 854 radiomic features from each segmentation, which we narrowed down to 29 via feature selection. We then combined these features with the Tumor, Node, Metastasis (TNM) system staging parameters, as well as the patient’s age. We trained a random survival forest model to perform an overall survival prediction over time, as well as a random forest classifier for the binary classification of two-year survival, using repeated cross-validation for evaluation. Our results exhibited promise, with a mean C-index of 0.731 for survival modeling and a mean accuracy of 0.76 in two-year survival prediction, providing evidence of the feasibility and potential efficacy of a fully automated pipeline for PDAC prognostication. By eliminating the labor-intensive manual segmentation process, our streamlined pipeline demonstrates an efficient and accurate prognostication process, laying the foundation for future research endeavors.

## 1. Introduction

Pancreatic ductal adenocarcinoma (PDAC), often used interchangeably with the term “pancreatic cancer”, is a leading cause of cancer-related mortality worldwide. As of 2023, it ranks as the third leading cause of cancer deaths in the United States, with an estimated 64,050 new diagnoses and 50,550 deaths [1]. Despite advances in detection and treatment methods, the five-year relative survival rate remains dismally low, at approximately 12.5% [1]. This dismal survival rate underscores the aggressive nature of the disease and the pressing need for more accurate and timely prognostic models.

Clinical features of PDAC often include weight loss, pruritus, jaundice, pain, dyspepsia, and new onset diabetes, amongst others [2]. However, the lack of early warning signs and the often delayed diagnosis contribute to the poor prognosis associated with the disease [3,4].

Computed tomography (CT) currently stands as the gold standard for the initial evaluation of suspected pancreatic cancer, with its sensitivity having improved up to 95% with advancements in scanner technology, scan protocols, and post-processing techniques [5,6]. Despite these strides, CT scans still face significant limitations, particularly in the realm of prognostication. Specifically, CT scans can provide staging information but do not offer significant prognostic insights, nor can they guide therapy beyond initial staging [7].

In recent years, several studies have explored the use of radiomic features for the prognostication of PDAC, demonstrating promising results [8]. These features, extracted from medical imaging, capture tumor characteristics that are not readily appreciated by the human eye, thereby potentially providing additional prognostic value. However, extracting these features from pancreatic tumors requires their manual delineation by trained clinicians, a labor-intensive and time-consuming process. This requirement forms a significant barrier to the wider adoption of those techniques in clinical practice.

In this study, we address these challenges by proposing an automated prognostic model for PDAC that employs pre-operative CT scans in conjunction with machine learning algorithms. This model automates the segmentation of the pancreas and associated tumors, leading to a subsequent prediction of patient survival predicated on a set of both radiomic and clinical features derived from the tumor and remaining pancreatic region. Importantly, we conduct an evaluation of the prognostic significance of the extracted features, individually for the tumor and the remaining pancreas. This approach streamlines the procurement of radiomics from both the tumor and the residual pancreas, circumventing the need for manual intervention.

In summary, our work makes the following contributions:

1. We propose and evaluate a fully automated pipeline for PDAC prognostication, effectively circumventing the need for manual delineation of the pancreas and tumor.
2. We assess the prognostic significance of radiomic features, derived from both the tumor region and the remaining pancreas, within our study cohort.
3. We validate our model’s capability to predict two-year survival in PDAC patients, thereby providing a tangible, clinically meaningful endpoint.

This advancement signifies a considerable enhancement over current practices, potentially empowering clinicians to formulate more informed treatment decisions and improve patient outcomes.

## 2. Materials and Methods

### 2.1. Dataset

**Figure 1:**
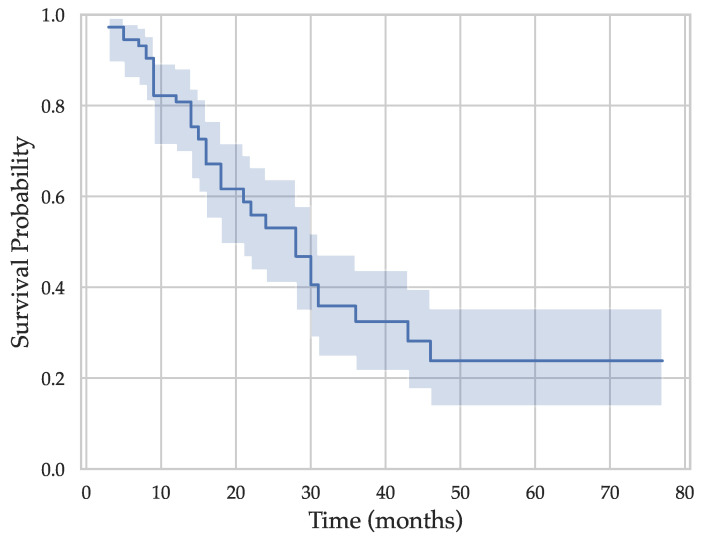
Kaplan–Meier survival curve calculated for 40 PDAC patients following pancreatectomy at Aretaieion Hospital.

The dataset used in this study includes pre-operative CT scans of 40 patients who underwent pancreatectomy for pancreatic cancer at Aretaieion Hospital in Athens, Greece (Ethics Committee approval code 327/9 April 2021). A standard pancreatic CT imaging protocol was followed for all patients (see Table 1). The cohort distribution was 24 male and 16 female patients, with a median age of 70 years (range 41–82 years). Histological analysis, conducted post-procedure, confirmed the presence of PDAC in all patients. Neoadjuvant chemotherapy was administered to two patients (5%), whereas all patients received adjuvant chemotherapy. The surgical procedure breakdown was as follows: 32 patients (80%) underwent pancreatoduodenectomy (Whipple’s procedure), 6 patients (15%) distal pancreatectomy with splenectomy, and 2 patients (5%) total pancreatectomy with splenectomy. Survival analysis revealed that 11 patients (27.5%) reached the end of the study period (right censored data). Figure 1 depicts the Kaplan–Meier survival curve for the cohort. The median survival time was 24 months.

### 2.2. Pipeline Architecture Overview

**Figure 2:**
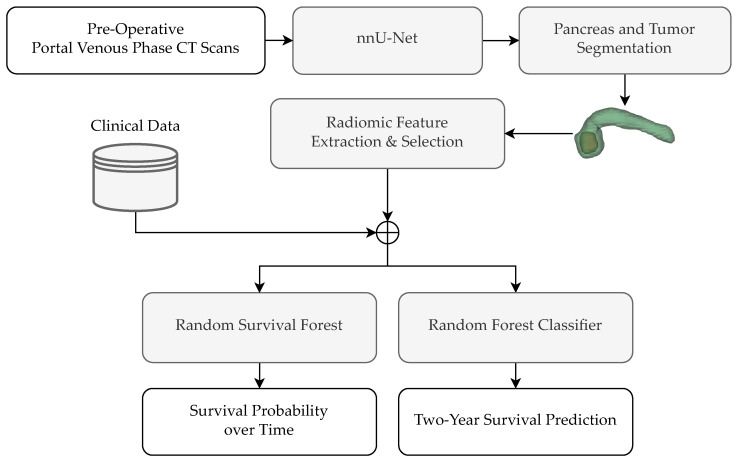
Architecture of the proposed pipeline.

An architectural overview of the pipeline investigated in this work is depicted in Figure 2.

First, for each individual patient, their pre-operative portal venous phase CT scan is processed by nnU-Net, a self-configuring neural network architecture that has demonstrated superior performance in medical image segmentation tasks [9]. The nnU-Net automatically identifies and delineates the pancreas and the tumor, producing a segmentation map that includes both regions. To this end, the nnU-Net was first trained on an external, publicly available dataset of PDAC patients [10]. Section 2.3 further details the segmentation step.

Following segmentation, radiomic features are extracted individually for the pancreas and tumor regions using the PyRadiomics library [11] (see Section 2.4). This is conducted by selecting the set of voxels from the original CT image, which have been labeled as either “pancreas” or “tumor” in the segmentation map. The radiomic features are then computed on each voxel set separately, capturing a wide range of characteristics related to intensity, texture, and shape.

Feature selection, detailed in Section 2.5, is then employed to reduce data dimensionality and enhance model performance. To this end, the multicollinearity among the computed radiomic features is assessed during training phase to reduce feature redundancy. Thus, only the features that were identified as the most relevant in the training set are kept. Subsequently, the subset of radiomic features is combined with clinical data, namely, the tumor staging and the patient’s age.

Finally, two different machine learning models are employed depending on the task (Section 2.6 and Section 2.8). For modeling the patient’s survival probability function over time, the random survival forests method is used [12]. On the other hand, for predicting the binary problem of the patient’s two-year survival, a random forest classifier is used.

### 2.3. Pancreas and Tumor Segmentation

To automatically extract precise segmentation maps for both the pancreas and tumor regions, we employed nnU-Net [9]. This network was selected due to its simplicity and superior performance, as evidenced by its state-of-the-art results in the Medical Segmentation Decathlon (MSD) [10], which encompasses diverse biomedical image analysis challenges, including the segmentation of tumors within the pancreas. Notably, the nnU-Net was the top-performing method on the Pancreas task, achieving a Dice Similarity Coefficient (DSC) of 0.80 and 0.52 for pancreas and tumor segmentation, respectively. The premise behind nnU-Net is that no new network architecture is necessary; instead of developing a domain-specific network design, loss function, or training scheme, the nnU-Net leverages a plain U-Net (hence the name “no new net”) and carefully selected pre-processing schemes. Further summary of the nnU-Net is included in Appendix B.

**Figure 3:**
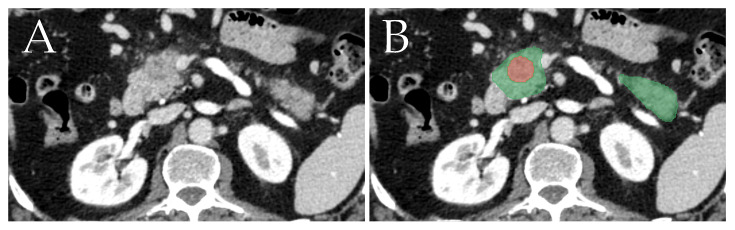
Example showcasing the segmentation of pancreatic ductal adenocarcinoma (red) and the rest of the pancreas (green). Image (**A**) depicts the original image, and image (**B**) depicts the image with the segmentations overlaid on top.

We trained the nnU-Net to segment the pancreas and tumors by using the 282 training samples provided by MSD. Then, the inference was ran on our dataset, and the produced pancreas-tumor masks were saved to be used for feature extraction. Figure 3 depicts an example of a segmentation result on a single image slice.

To ensure optimal data quality and consistency, we followed the pre-processing protocol described in [9]. Initially, the median spacing (in millimeters) was computed across all CT scans, and all data were resampled accordingly using linear interpolation. Subsequently, contrast stretching was performed by clamping all CT intensity values within the bottom 0.5% and top 99.5% range. Finally, all scans were normalized by subtracting the mean and dividing by the standard deviation, which were again computed based on the entire dataset.

### 2.4. Feature Acquisition

In the feature extraction step, we employed radiomics, a quantitative image analysis method that extracts a large number of advanced features from medical images [13]. When applied on a segmented tumor region, the radiomic features capture various characteristics related to its intensity, texture, and shape. These features have previously demonstrated promise in predicting treatment response and outcomes, tumor staging, and tissue identification, among others [14,15,16].

In our study, we used the PyRadiomics library [11], an open-source python package that provides a standardized and efficient way to compute radiomic features from medical images. This package handles loading both the image and the corresponding segmentation map, filtering (for example wavelet filters), and feature calculation, offering a streamlined approach that serves as a reference standard for radiomics, ensuring reproducibility and comparability. In total, 854 radiomic features were extracted from each pancreas and tumor segmentation. These features included first order features, shape features, Grey Level Co-occurence Matrix (GLCM) features, Grey Level Size Zone Matrix (GLSZM) features, Grey Level Run Length Matrix (GLRLM) features, Neighbouring Grey Tone Difference Matrix (NGTDM) features, and Gray Level Dependence Matrix (GLDM) features, computed on the original and wavelet-filtered images.

### 2.5. Feature Selection

The extraction of 854 radiomic features from both the pancreas and tumor segmentations creates an expansive and high-dimensional feature space, far exceeding the total number of samples in the dataset. This phenomenon, often referred to as the “curse of dimensionality”, introduces challenges in building accurate predictive models due to data sparsity. The abundance of features poses a risk of the model capturing noise and irrelevant patterns instead of true relationships, leading to overfitting and generalization issues. To address these challenges and improve the model’s performance, a vital pre-processing step involves feature selection. This process aims to identify the most informative features that significantly contribute to the model’s predictive power while discarding irrelevant or redundant ones.

To this end, we employed the Variance Inflation Factor (VIF) technique to assess multicollinearity among the extracted radiomic features. More specifically, the VIF measures how much the variance of an estimated regression coefficient increases when a particular predictor variable is included in a linear regression model, relative to when that predictor variable is excluded. A high VIF value for a specific variable indicates high linear correlation with other variables.

We iteratively computed the VIF values for all radiomic features. After each iteration, the feature with the highest VIF value was removed from the dataset and the analysis was re-ran. The iterative process continued until the highest observed VIF value fell below 10, signifying reduced multicollinearity. By applying this technique, the number of features were effectively narrowed down to a more manageable set of 29. The list of selected radiomic features can be found in Appendix A, Table A1. This streamlined feature selection approach ensures that the selected radiomic features capture the most relevant information for our predictive model.

In addition to the selected radiomic features, we added clinical features, namely, the TNM classification of the tumor [17] expressed as three distinct integers valued between 0–3 and the patients’ age in years. Thus, the total number of features amounted to 33.

### 2.6. Survival Modeling

In this study, we employed the random survival forests method proposed by Ishwaran et al. [12] to model the survival probability function for each patient based on their respective radiomic and clinical features. The random survival forests method is an ensemble tree-based method designed for analyzing right-censored data. The rationale behind choosing random survival forests over more traditional methods, such as Cox’s proportional hazards model [18], lies in its ability to capture non-linear relationships between predictors and survival outcomes, while not making the assumption of proportional hazards. This attribute renders it particularly well-suited for scenarios involving complex interactions between features and survival times.

In order to assess the prognostic value of the extracted features, we partitioned the dataset into two distinct groups: one composed of tumor features, and the other involving features from the remaining part of the pancreas. Consequently, we conducted separate investigations into the predictive capability of the model for each set of features, as well as their combination. To facilitate a more accurate evaluation on our limited dataset, we adopted a 5-fold cross-validation procedure. We aggregated all predictions as calculated over all folds and computed the Harrell’s Concordance Index (C-index) [19]. To obtain an estimate of the amount of variability due to data shuffling, we repeated the process 10 times, each time randomly re-partitioning the data into the 5 folds. We report the final C-index as the mean value and the standard deviation over the 10 repetitions. The model’s hyperparameters were set heuristically as follows: we set the number of trees to 100, the maximum number of features to consider for each split as the base-2 logarithm of the total number of features, the minimum number of samples for a leaf node to 5, and the minimum number of samples needed to split a node to 2. Regarding the tree depth, we allowed the nodes to expand until all leaves contained fewer than 2 samples.

### 2.7. Feature Importance Analysis

To evaluate the importance of the selected radiomic and clinical features in predicting patient survival, we conducted a feature importance analysis using the permutation importance method [20]. The aim was to evaluate the impact of individual features on the predictive performance of the random survival forests model.

The permutation feature importance is defined as the decrease in a model’s performance when the values of a single feature are randomly shuffled while keeping all other features unchanged [20]. By permuting the values of a specific feature, we break the relationship between that feature and the target variable, and the resulting drop in the model’s performance provides insight into the feature’s importance.

In order to provide a baseline for comparison, we introduced a single noise feature, randomly drawn from a Gaussian distribution with a mean of 0 and a standard deviation of 1. By assessing the importance of other features in relation to the noise feature, we can quantify their relative contributions to the model’s predictive performance.

With the noise feature added to the dataset, we re-trained the random survival forests model on the modified data. The model’s hyperparameters and training scheme were set as specified in Section 2.6. For each feature in the dataset, we computed its permutation importance by randomly permuting the feature’s values across all samples, while keeping the other features fixed. The model’s performance was then evaluated on each fold using the C-index. The difference between the original C-index and the C-index with shuffled data quantified the importance of each feature relative to the noise feature. To obtain robust estimates of feature importances, we computed the mean and standard deviation of each feature’s importance value across all folds.

### 2.8. Two-Year Survival Classification

In addition to providing a survival probability curve over time, we investigated the ability of a Random Forest Classifier in predicting patients’ two-year survival. To this end, we set the model’s hyperparameters as in Section 2.6. We trained two classifiers, each on the features originating from the tumor and the rest of the pancreas, respectively, using 5-fold cross-validation and reported the metrics over the aggregated predictions set.

## 3. Results

### 3.1. Survival Modeling

We tested the random survival forests separately on features extracted from the tumor and the rest of the pancreas, computing the C-index over all predictions using 5-fold cross-validation, repeated 10 times. For the tumor features, we computed a C-index of 0.731 ± 0.015. For the features from the rest of the pancreas, the achieved C-index was 0.485 ± 0.041.

### 3.2. Feature Importance Analysis

**Figure 4:**
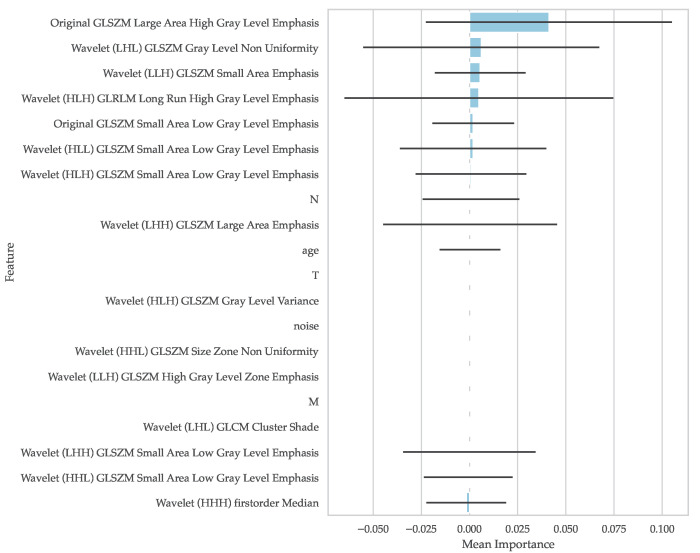
Top 20 features by mean importance.

Figure 4 depicts a visual comparison of the importances of the top 20 features by their mean permutation importance, expressed as the decrease in the C-index whenever a particular feature was randomly shuffled.

Overall, the Large Area High Gray Level Emphasis (LAHGLE) on the original image type far exceeded the rest of the features in importance, with a value of 0.041. In comparison, the Gray Level Non Uniformity on the wavelet (LHL) image came in second, with an importance of 0.006. Noise, which was inserted as a reference value, manifested an importance of zero.

### 3.3. Two-Year Survival Classification

Table 2 depicts the accuracy, recall, specificity, precision, and *F*1 score, as calculated over the 10-times repeated 5-fold cross-validation. For this binary classification problem, a patient surviving beyond 24 months was considered a “positive” sample.

## 4. Discussion

### 4.1. Survival Modeling

In the context of overall survival modeling, our tests on the random survival forests were conducted separately on features derived from the tumor and the remainder of the pancreas. The methodology involved computing the C-index over all predictions using a 5-fold cross-validation, repeated 10 times. The tumor features returned a mean C-index of 0.731, considerably higher than the 0.485 garnered from the features of the rest of the pancreas. This divergence indicates the relative importance and predictive power of the tumor features for PDAC prognostication.

Furthermore, the observed C-index of 0.731 indicates that the fully automated pipeline proposed in the present study could potentially provide a superior prognostic alternative compared to TNM staging. This is evident when considering the research conducted by Chen et al. (2016) [7], where the prognostic efficacy of the staging characteristics from the 8th edition of the AJCC cancer staging manual [21] was evaluated, finding a C-index of 0.585. Similarly, in a study conducted by Mohammad et al. (2023) [22], the observed C-index was found to be 0.633.

There has been a prevalence of studies reporting improved performance with the use of radiomics, a finding that aligns with our results. For instance, in a study by Park et al. (2021) [23], it was reported that the addition of radiomics to clinical features improved the C-index from 0.679 to 0.741. Similar results were reported by Xie et al. (2020) [24], who reported a C-index of 0.726 achieved through the use of radiomic features. Conversely, other investigations have documented lower *C*-indices with radiomics, even though these were superior to the results achieved solely through clinical features. Healy et al. (2022) [25] reported a C-index of 0.545 using clinical–radiomic features, while Zhang et al. (2020) [26] reported a C-index of 0.491 with radiomics, which was later improved to 0.651 when using learned features from a Convolutional Neural Network.

The discrepancies among these studies’ results may be attributed to several variables, including diversity in feature selection methods, variations in feature pre-processing techniques, and inherent heterogeneity in the datasets used. However, the alignment of our results with the top-performing studies offers compelling evidence for the feasibility of fully automating the prognostic process for PDAC. This is especially important when considering that all studies so far depend on manual segmentation of the tumor and pancreas.

### 4.2. Feature Importance Analysis

The graphical depiction of feature importances in Figure 4 revealed that only a limited number of features are integral to survival estimation. The LAHGLE on the original image type stood out as the most significant feature, with an importance value of 0.041. This measure vastly exceeded the second most important feature, the Gray Level Non Uniformity on the wavelet (LHL) image, which demonstrated a low importance of 0.006.

The LAHGLE measures the proportion in the image of the joint distribution of larger zones with higher gray level values [27]. Thus, it appears that survival probability may be, at least partially, associated with the extent of the tumor composed of large patches of contiguous, high-intensity pixels. The specific directionality of this association remains undetermined, given the calculated feature importance does not delineate whether a higher survival probability correlates with a higher or lower LAHGLE value.

An application of the Cox proportional hazards model [18] using only the LAHGLE feature yielded a C-index of 0.682 ± 0.014. While this index does not match that obtained using all available features, it further underscores the predictive significance of this particular feature.

Furthermore, the importance analysis appears to suggest that the TNM classification bears limited relevance to the prediction. However, it should be noted that this relevance is dependent upon the specific characteristics of the dataset, which in this study demonstrated minimal heterogeneity with respect to TNM classification. For instance, no patients in the cohort exhibited metastasis (M was 0). Moreover, it is crucial to recognize that the identified importance of the features is not an absolute measure of their association with survival. Rather, it is specific to the predictive model utilized in this study, the random survival forests, as well as the employed segmentation network. Therefore, the identified feature importances might vary with the use of other predictive models or segmentation approaches.

### 4.3. Two-Year Survival Classification

As depicted in Table 2, features derived from tumor tissue markedly outperformed those obtained from the rest of the pancreas. The tumor features exhibited an *F*1 score of 0.74 ± 0.04, accuracy of 0.76 ± 0.04, and a precision of 0.75 ± 0.04. Conversely, the performance metrics for features derived from the remaining pancreatic tissue were substantially lower. This finding underscores the significance of prioritizing tumor-specific features, which appear to bear enhanced predictive capability compared to features drawn from the remainder of the pancreas.

Our results concur with analogous studies examining the prediction of two-year survival using radiomic features extracted from manual segmentations. Specifically, the study conducted by Osman (2019) [28] reported a recall of 0.79 and an area under the curve (AUC) of 0.82. Meanwhile, the investigation by Chakraborty et al. (2017) [29] documented an accuracy of 0.75, recall of 0.68, and specificity of 0.80. This alignment of results further validates our approach and reinforces the validity of utilizing an automated pipeline for prognostication.

### 4.4. Limitations

This study has two limitations that should be acknowledged.

Firstly, the validation of our automated approach was performed on a limited dataset of 40 patients from a single institution. While we performed rigorous testing using repeated cross-validation and computed our results over the entire dataset size, it is possible that the generalizability of the findings is limited across different clinical settings and patient populations. Future research should aim to validate these findings in a larger, multicenter dataset, which would provide a more robust test of the system’s effectiveness.

Secondly, the fully automated nature of our pipeline implies that the quality of segmentation directly impacts the performance of the downstream radiomics analysis. Although nnU-Net has demonstrated satisfactory segmentation results, it should be noted that any inaccuracies in the segmentation process can lead to suboptimal feature extraction, which might, in turn, impact the performance of the survival and binary classifiers.

### 4.5. Future Perspectives

Future studies could leverage this automated pipeline and identified prognostic features to guide treatment decisions according to predicted patient outcomes. This could range from a more aggressive approach in patients predicted to have a more favorable prognosis to a more conservative focus in those predicted to have a less favorable outcome (or vice versa).

## 5. Conclusions

In the present study, we investigated the feasibility of employing a fully automated pipeline for PDAC prognostication. This pipeline incorporated a 3D Convolutional Neural Network, specifically, nnU-Net, initially to segment the pancreas and the tumor. Based on these segmentations, pertinent radiomic features were extracted and subsequently combined with clinical data, specifically, the patients’ ages and TNM tumor classification. Employing random survival forests, an estimation of the patients’ survival probability over time was conducted. In addition, a random forest classifier was utilized for addressing the binary classification issue of predicting two-year survival.

The study outcomes have demonstrated that the fully automated pipeline delivers a promising performance for PDAC prognostication. Specifically, the approach exhibited superior performance metrics compared to existing methods solely reliant on clinical variables such as TNM staging. Moreover, the findings of this study were consistent with similar studies in which manual segmentations were employed, providing an indication of the feasibility and potential efficiency gains of a fully automated approach. The results also highlighted the critical importance of specific features, notably, the LAHGLE, further signifying its predictive value for survival estimation.

In conclusion, our research has provided evidence of the feasibility and potential efficacy of a fully automated pipeline for PDAC prognostication. However, it necessitates further investigation and validation across larger and more diverse datasets to substantiate its generalizability and robustness. With further advancements, this approach could potentially promote more accurate, efficient, and individualized patient care.

## Figures and Tables

**Table 1:** Pancreatic CT imaging protocol detailing the recommended utilization of CT scan as the primary diagnostic technique for comprehensive evaluation of patients with pancreatic cancer.

Parameter	Details
Scan type	Helical
Section thickness	Preferably submillimeter (0.5–1.0 mm)
Interval	Same as section thickness
Oral contrast agent	Neutral or low-Hounsefield units
Intravenous contrast agent	Preferably high iodine concentration (>300 mg I/mL) at an injection rate of 3–5 mL/s
Scan acquisition	Pancreatic parenchymal phase at 40–50 s; portal venous phase at 65–70 s
Image reconstruction	Axial 2–5 mm thickness Multiplanar reformats in the coronal plane at 2–3 mm thickness, and per institutional preference, the sagittal plane Maximum intensity projections or three-dimensional volumetric thick sections for vascular evaluation

**Table 2:** Performance metrics for the binary classification problem. Patients who survived beyond 24 months were considered positive samples.

Feature Origin	F1 Score	Accuracy	Specificity	Recall	Precision
Tumor	0.74 ± 0.04	0.76 ± 0.04	0.78 ± 0.04	0.74 ± 0.05	0.75 ± 0.04
Rest of the pancreas	0.48 ± 0.07	0.51 ± 0.06	0.54 ± 0.08	0.47 ± 0.07	0.50 ± 0.06

## Data Availability

The data presented in this study are available upon request from the corresponding author dependent on ethics board approval. The data are not publicly available due to data protection legislation.

## Appendix A

**Table A1:** Complete list of the 37 features after feature selection with iterative Variance Inflation Factor (IVF).

Image Type	Feature Class	Feature
Original	GLDM	Small Dependence Emphasis
Original	GLRLM	High Gray Level Run Emphasis
Original	GLSZM	Large Area High Gray Level Emphasis
Original	GLSZM	Small Area Low Gray Level Emphasis
Wavelet (LLH)	First Order	Total Energy
Wavelet (LLH)	GLSZM	Gray Level Non-Uniformity
Wavelet (LLH)	GLSZM	High Gray Level Zone Emphasis
Wavelet (LLH)	GLSZM	Small Area Emphasis
Wavelet (LLH)	GLSZM	Zone Entropy
Wavelet (LHL)	GLCM	Cluster Shade
Wavelet (LHL)	GLCM	Imc2
Wavelet (LHL)	GLSZM	Gray Level Non-Uniformity
Wavelet (LHH)	GLCM	Difference Entropy
Wavelet (LHH)	GLDM	Low Gray Level Emphasis
Wavelet (LHH)	GLRLM	Gray Level Variance
Wavelet (LHH)	GLSZM	Large Area Emphasis
Wavelet (LHH)	GLSZM	Small Area Low Gray Level Emphasis
Wavelet (HLL)	First Order	Kurtosis
Wavelet (HLL)	GLSZM	Size Zone Non-Uniformity
Wavelet (HLL)	GLSZM	Small Area Low Gray Level Emphasis
Wavelet (HLH)	GLRLM	Long Run High Gray Level Emphasis
Wavelet (HLH)	GLSZM	Gray Level Variance
Wavelet (HLH)	GLSZM	Small Area Emphasis
Wavelet (HLH)	GLSZM	Small Area Low Gray Level Emphasis
Wavelet (HHL)	GLSZM	Size Zone Non-Uniformity
Wavelet (HHL)	GLSZM	Small Area Low Gray Level Emphasis
Wavelet (HHH)	First Order	Median
Wavelet (LLL)	GLRLM	Long Run Low Gray Level Emphasis
Wavelet (LLL)	GLSZM	Gray Level Non-Uniformity

## Appendix B

The nnU-Net is a semantic segmentation method proposed by Isensee et al. [9] that automatically configures a U-Net [30] by adapting to each particular dataset without manual intervention. Its prowess was notably demonstrated in the Medical Segmentation Decathlon [10], where it competed with other segmentation pipelines on 23 datasets, surpassing most of them and scoring several first places.

The U-Net is a Convolutional Neural Network (CNN) architecture with a U-shaped architecture, hence its name [30]. Its design combines a contracting path, which captures image context and reduces its spatial dimensions through a series of convolutional and pooling layers, with an expansive path that enables precise localization through up-sampling and transposed convolutions. U-Net’s distinctive feature is the incorporation of skip connections that transfer high-resolution features from the contracting path to the extracting path, aiding in the preservation of spatial information during up-sampling.

The nnU-Net uses three different U-Net configurations: a 2D U-Net, a 3D U-Net, and a 3D U-Net cascade. In the cascade, the first U-Net processes downsampled images, and the second U-Net refines the segmentation maps from the first U-Net at full resolution. Through cross-validation, nnU-Net identifies the most effective configuration or ensemble.

Prior to training, the nnU-Net pipeline defines a dataset fingerprint encompassing image modality, shape, voxel spacing, and intensity distribution. This fingerprint guides overall network design and hyperparameter selection. The nnU-Net pre-processes the data accordingly by resampling the image and transforming the voxel spacing, as well as normalizing its intensity values. Then, design choices are made depending on the computed dataset fingerprint and the machine’s hardware specifications. For example, the patch size to be used during training is directly affected by the input image size, number of mini-batches, and the available GPU memory. The nnU-Net automatically selects the patch size and the number of mini-batches to achieve high performance, while making effective use of all the available resources.

Therefore, the nnU-Net’s strength lies in its simple underlying architecture (the U-Net) and its automated adaptation to diverse datasets, choice of optimal configurations, and efficient use of available resources.
